# INTERDISCIPLINARY PERSPECTIVES ON THE INTERSECTION OF CANCER AND COGNITIVE DECLINE

**DOI:** 10.1093/geroni/igad104.0088

**Published:** 2023-12-21

**Authors:** Judith Carroll, Lauren Stratton, Brent Small

## Abstract

The population of older cancer survivors in the United States is rapidly growing. The long-term cognitive aging of older cancer survivors is not well understood, as large-scale epidemiological studies often show a paradoxical inverse association between cancer history and subsequent dementia; yet cancer survivors often experience cognitive impairment symptoms in the short-term following treatment. Further, the cancer diagnosis and care experiences of older adults experiencing cognitive impairments or dementia warrant further investigation as these are important issues for health care quality and equity amongst older adults experiencing cognitive limitations who are at risk for cancer. The novel findings presented in this interdisciplinary symposium will help to clarify the cognitive aging experiences of older cancer survivors, using innovative data sources. First, Brown will discuss the relationship between cancer and subjective cognitive decline, using the nationally representative Behavioral Risk Factor Surveillance System data. Second, Westrick will present the associations between cancer treatment types and subsequent mortality and cognitive decline for survivors of breast, prostate, and kidney cancer among Medicare beneficiaries in the nationally representative US Health and Retirement Study. Third, also using data from Medicare beneficiaries in the US Health and Retirement Study combined with a mixed-methods physician survey, Mullins will discuss differences in cancer treatment recommendations and receipt for older adults with a pre-existing cognitive impairment who are diagnosed with cancer. Finally, Todorova will take a deeper dive into the potential mechanisms linking cancer treatment with cognitive decline, by examining a biomarker of inflammation and cardiovascular toxicity among patients receiving doxorubicin. This is a collaborative symposium between the Alzheimer’s Disease and Related Dementias and Cancer and Aging Interest Groups.

